# Ontology-Based Data Integration between Clinical and Research Systems

**DOI:** 10.1371/journal.pone.0116656

**Published:** 2015-01-14

**Authors:** Sebastian Mate, Felix Köpcke, Dennis Toddenroth, Marcus Martin, Hans-Ulrich Prokosch, Thomas Bürkle, Thomas Ganslandt

**Affiliations:** 1 Institute for Medical Informatics, University Erlangen-Nuremberg, Erlangen, Germany; 2 Center for Medical Information and Communication, Erlangen University Hospital, Erlangen, Germany; 3 Tumor Centre, Erlangen University Hospital, Erlangen, Germany; 4 Institute for Medical Informatics, Bern University of Applied Sciences, Bern, Switzerland; Gentofte University Hospital, DENMARK

## Abstract

Data from the electronic medical record comprise numerous structured but uncoded ele-ments, which are not linked to standard terminologies. Reuse of such data for secondary research purposes has gained in importance recently. However, the identification of rele-vant data elements and the creation of database jobs for extraction, transformation and loading (ETL) are challenging: With current methods such as data warehousing, it is not feasible to efficiently maintain and reuse semantically complex data extraction and trans-formation routines. We present an ontology-supported approach to overcome this challenge by making use of abstraction: Instead of defining ETL procedures at the database level, we use ontologies to organize and describe the medical concepts of both the source system and the target system. Instead of using unique, specifically developed SQL statements or ETL jobs, we define declarative transformation rules within ontologies and illustrate how these constructs can then be used to automatically generate SQL code to perform the desired ETL procedures. This demonstrates how a suitable level of abstraction may not only aid the interpretation of clinical data, but can also foster the reutilization of methods for un-locking it.

## Introduction and Background

Reusing clinical routine care data in single source projects [1] has gained in importance recently [2–5]. The data are used for feasibility studies, patient recruitment, the execution of clinical trials [6–10], clinical research [11–14] and biobanking [15].

Routine care data can roughly be classified into three categories: *(1) unstructured free text,* which is used for flexible documentation items such as discharge letters, clinical notes and findings, *(2) structured and coded data elements,* which are coded according to standardized terminologies and are typically used for billing and *(3) structured but uncoded data elements,* which are used in assessment forms of electronic medical records (EMRs).

The first type, unstructured free text, provides the most comprehensive information, because it does not restrict the clinical user during the documentation process [16]. An automated analysis, however, requires complex *natural language processing* methods [17]. The second type, structured and coded data, is easier to process, but is limited in terms of expressiveness and credibility [18, 19]. In this manuscript we concentrate on structured but uncoded data, the third type. It encodes information with enumerable value lists that are, however, not linked to any standard terminology. Many EMR forms comprise such data. Its reuse is challenging due to the following four reasons:

- **It is difficult to process non-standardized data elements:** Assessment forms are often designed to mimic classic paper sheets. They are typically used to record events during the hospitalization, e.g. the medical history, examinations, surgical procedures and different pathological findings and are often highly customized. According to [20], assessment forms *“[ …] have been developed to fulfill the specific requirements of the hospital unit, and the data is described according to the definitions of assessments and concepts that are used locally”*. Thus, the value sets are often not linked to standard classifications. For example, the data element ‘sex’ might be encoded with the value set {female, male} in one form and with {F, M} or {1, 2} in another. Although some of these concepts could be separately linked to standard terminologies (in the example we could link {female, male} to the standardized SNOMED-CT [21] codes {248152002, 248153007}), many other value lists are use-case-specific and cannot be mapped.
- **EMRs lack knowledge management functions:** Most EMR systems do not offer data dictionaries [22] with clear concept definitions to enable the reuse of data elements in multiple forms [23], although their advantages have been known for a long time [24]. Instead of defining and reusing concepts such as *weight, height* or *smoker status*, these elements are frequently redefined for each form. Over time, this results in an accumulation of inconsistent concept naming and value sets within new EMR forms and complicates data extraction and interpretation, because the redundant data elements have to be identified and merged.
- **Contextual semantic relationships between data elements and forms are lost:** A clinical user considers the medical context, the structure of the assessment form and the neighboring data elements when entering new data. He would, for example, understand a TNM [25] documentation field in a pathology form as a *pathological TNM* and not as a *clinical TNM*. The TNM is a classification to describe a patient’s cancer status in terms of tumor size, affected lymph nodes and metastases. However, such implicit relationships are not stored in most clinical systems. When extracting the data, the data engineer has to manually review the forms and remodel these semantic relationships in his database transactions.
- **It is a challenge to integrate data from different institutions:** Previous efforts to integrate data from different EMRs [26–31] demonstrated success, but also identified challenges if the semantic representation between the EMR sources differed. Merging data between a hospital EMR and a cancer registry record for instance turned out to be difficult, because EMR data was linked to the patient but the cancer registry distinguished between cancer treatment applied to the main tumor and treatment of metastases and recurrence [31]. Such problems resulted in large manual efforts spent for the data integration in recent cross-institutional research projects [26, 27, 30].

### Objective

Today a data engineer is required to address these challenges while preparing EMR data for reuse. The implicit knowledge gained in the extraction process, e.g. about data context and provenance, is conventionally not recorded in a universally machine-processible format and therefore is lost. The data extraction, transformation and loading (ETL) procedures are unique for each database system and cannot be reused. The complex ETL procedures are difficult to understand and to maintain.

Our goal was to develop a method that is based on declarative, universally machine-processible but also human-readable and easily maintainable ETL definitions that can be translated into automated database transactions. Our approach incorporates the ETL know-how in an ontological system that governs the correct extractions and data transformations.

## Methods

### The EMR in Erlangen

The Erlangen University Hospital is a 1,360-bed tertiary care unit in southern Germany. It deployed the EMR system Soarian Clinicals by Siemens [32] in 2003. In the following years, the system was rolled out in all clinical specialties for order entry, results reporting as well as medical and nursing documentation. Today, the EMR is used in more than 90 wards, in functional units such as echocardiography and in outpatient clinics by more than 2,800 registered users. It supports the design of custom assessment forms and workflows for specialized purposes. Using this toolbox, extensive electronic documentation instruments had been established for many clinical specialties in recent years. For example, detailed assessment forms for prostate, mamma, thorax, and colorectal carcinoma have been provided to support patient care in the Erlangen comprehensive cancer center [33]. Today, the EMR comprises 785 different assessment forms, which contain 28,055 data elements with 35,301 distinct selectable values. The system stores data of approximately 1,150,000 patients.

Several projects at Erlangen University Hospital reuse structured but uncoded EMR data in cross-institutional research settings [33–35]. In these projects we were confronted with the problems described in the introduction.

**Figure 1:**
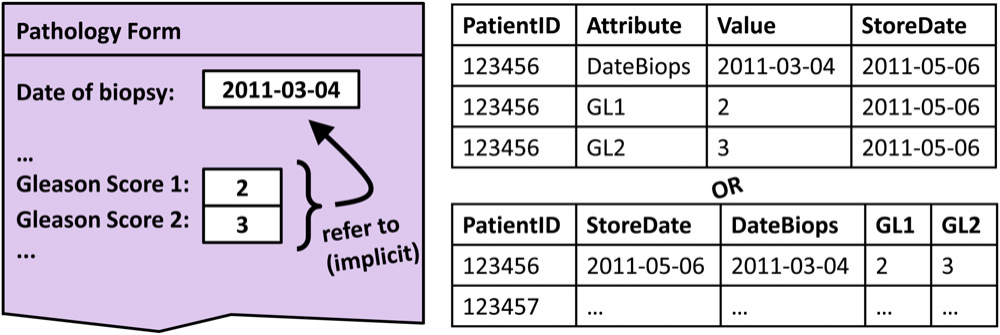
Generic databases do not reflect semantic relationships between data elements. The two (equivalent) database tables on the right side (EAV-like style on top, column-oriented style below) do neither reflect the form’s structure as it is visible to the application user (left side), nor the semantic relationships between different input elements.

Fig. 1 illustrates a typical example. The Gleason Score describes the microscopic appearance of prostate tumors. Cell differentiation of the most common and the second most common tumor pattern are rated on a five-point scale from grade 1 (well differentiated) to grade 5 (poorly differentiated). The sum of both is the *Gleason Score*. Each Gleason Score thus consists of three parts (e.g. 2 + 3 = 5), which are denoted as *Gleason Score 1*, *2* and *3* in the EMR. An additional date field stores the time stamp of the biopsy. The EMR database, however, does not store this relationship explicitly, but treats all data elements separately. Thus the scores are attributed with the storage date of the assessment form (2011–05–06), while according to the clinical meaning, the reference date should be the value of the biopsy date element (2011–03–04).

While this exemplary ETL task may be easy to solve, one must bear in mind that typical single source projects require dozens or even hundreds of patient characteristics. Thus, the identification of all relevant data elements from large EMRs with tens of thousands of data elements, their semantic harmonization and the continuous maintenance of this ETL is a Sisyphean struggle.

We now describe our ontology-based approach that aims to simplify and support the mapping, extraction and data transfer processes.

### Ontological representation of source and target systems and mappings

In a first step, we define the ETL process as a declarative representation that is stored in ontologies. In the scope of this paper we understand an ontology to be a directed graph. The graph’s nodes represent entities while the edges describe relationships between them. Two nodes that are connected via an edge are called a triple. We use the Semantic Web [36] standard *Resource Description Framework* (RDF) [37, 38], where nodes are termed *resources* and edges are termed *properties*. We also use some constructs from RDF Schema [39] and the Web Ontology Language (OWL) [40], although these are generously simplified for the readability of this paper and its illustrations. For example, we omit the distinction between classes and instances. However, this has no impact on the validity of our approach. The supplied appendix in S1 File distinguishes between classes and instances.

**Figure 2:**
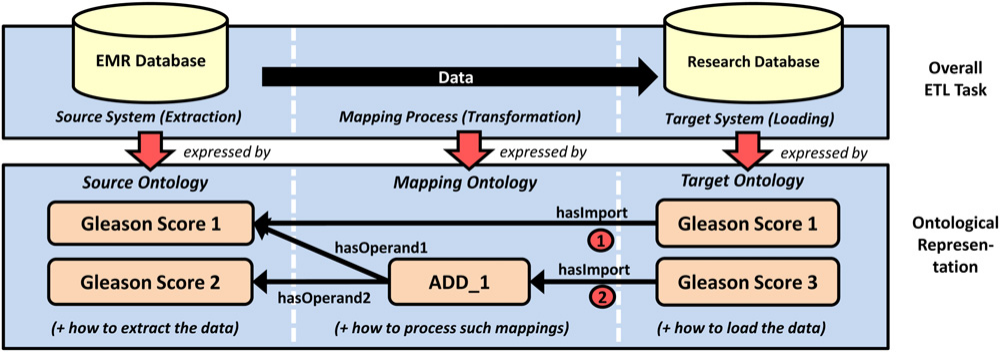
ETL steps are represented with ontologies. Components and processes involved in the extraction, transformation and loading of data are represented with ontologies. The mappings (1) and (2) illustrate “simple” and “complex” mappings, respectively.

The upper part of Fig. 2 shows a typical ETL process, where data records have to be extracted from a source system (EMR database, left), transformed (black arrow, center) and then loaded into a target system (research database, right). The lower part of the figure illustrates our abstraction approach with ontologies. Our key concept is to express each of the three ETL steps with an ontology: A *source ontology* corresponds to the extraction, whereas a *target ontology* corresponds to the loading. By creating connections between these two ontologies in a *mapping ontology*, the user defines how data is to be transformed between both. Later, the mapping ontology can be automatically translated into executable SQL transactions.

### The source ontology

The *source ontology* is used to describe the contents of the source system’s database. It serves two important functions. First, it acts as an inventory of all available medical data elements in the source system. These concepts can be organized in hierarchies to reflect the content structure of the source system. This allows the user to easily navigate the ontology and to select relevant concepts while creating the mappings.

Second, it provides an inventory-to-database-schema mapping by abstracting database record sets with ontology concepts. A source ontology concept *Gleason Score 1,* for example, represents the set of all Gleason Score 1 data in the source system. This record set is a list of all patient IDs, for which at least one *Gleason Score 1* is available. Additional columns for value and timestamp next to the patient IDs later allow comparisons and computations between multiple lists, e.g. it will become possible to sum the records of the lists “Gleason Score 1” and “Gleason Score 2” to derive a new “Gleason Score 3” list. We call this schema of three columns (PatientID, Value, Date) our *internal data model*.

**Figure 3:**
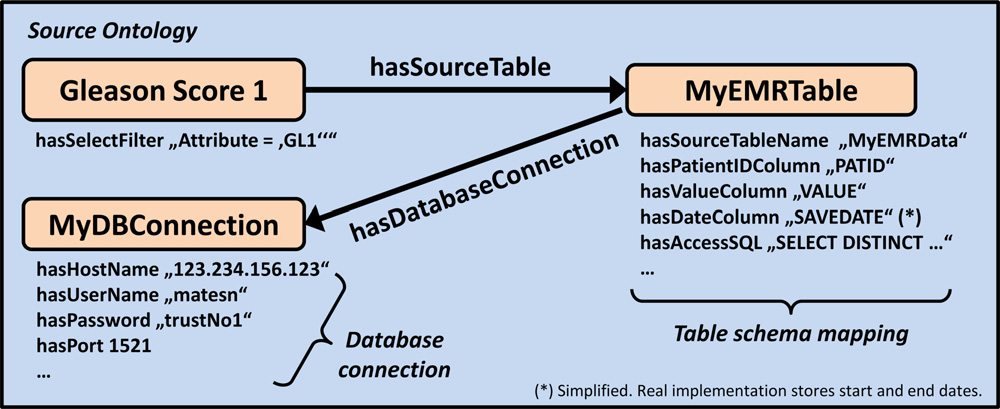
Database bindings are described with ontologies. By linking the medical concept “Gleason Score 1” to additional ontology concepts that describe the database schema and connection, a software component can construct SQL to retrieve the data records. *Note: To save space within the figures, datatype properties are printed below the concepts. These statements have to be read like this: MyEMRTable hasSourceTableName “MyEMRData”.*

Fig. 3 illustrates how this inventory-to-database-schema mapping is achieved using ontologies. The medical concept *Gleason Score 1* from the source ontology’s “inventory” is connected to a table instance *MyEMRTable* with a *hasSourceTable* relationship, which is linked to a database connection instance *MyDBConnection*. These instances use RDF datatype properties (i.e. string values) to store information about the database connection and the table schema. A software component can process this information and map the source database schema to the internal data model. The source table column *PATID* from the source system for example is translated to the *PatientID* column of the internal data model using the statement *MyEMRTable hasPatientIDColumn “PatID”*. Respectively, the properties *hasValueColumn* and *hasDateColumn* provide the mappings to the columns *Value* and *Date* in the internal data model.

Some clinical EMR systems, such as Siemens Soarian [32] or Epic [41], store data in an entity-attribute-value format [42, 43]. In this case additional filter criteria are necessary to retrieve only the data records that are associated with the desired concept. They are implemented with *hasSelectFilter* datatype properties, which are connected to each source ontology concept (see Fig. 3, below the *Gleason Score 1* concept). We can now construct an SQL statement that returns all desired Gleason Score 1 records in the internal data model:

SELECT DISTINCT **PATID** PatientID, **VALUE** Value, **SAVEDATE** Date

FROM **MyEMRData** WHERE **Attribute = ‘GL1’**

The underlined parts are retrieved from the ontologies (see Fig. 3) and inserted into an SQL template, which is also stored in the ontology (*hasAccessSQL* property). Different templates can be created to access different database schemas. This template-based SQL code generation is used throughout our approach to achieve the necessary database bindings.

### The target ontology

The *target ontology* is similar to the source ontology, but used for loading instead of extracting data. It represents a domain ontology, because it describes the collection of medical concepts to be loaded into the target system (the target dataset). In cross-institutional settings where multiple sites share their data in a central research database, the target dataset has to be defined before the creation of the target ontology. Such data elements are also called *common data elements* [44, 45].

The target ontology contains syntactic and semantic information that is linked to each concept and is used to generate the metadata for the target system. For demonstration purposes we chose *Informatics For Integrating Biology And The Bedside* (i2b2) [46], an open source research platform that can be used to identify patient cohorts, as an exemplary target system. Therefore, the target ontology has to implement the semantic features of i2b2. These include, for example, the data type of the concept, a short textual description, and, if applicable, the unit of measurement for numeric values and further attributes such as lab value ranges. Tables A-D in S1 File list all ontology constructs.

### The mapping ontology

**Figure 4:**
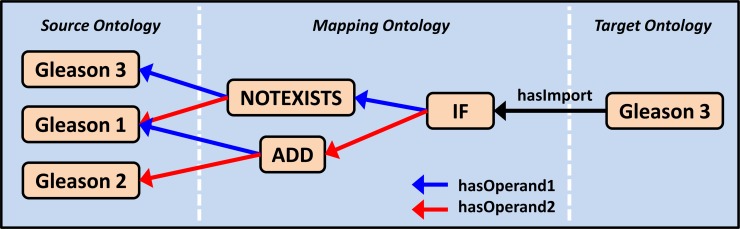
Cascading of mapping nodes. Cascaded mapping nodes allow the definition of arbitrary data transformations. The illustration has to be read from the right to the left, hasOperand1 before hasOperand2. Paraphrased, it means: If no data for Gleason 3 (left side) exist, add the Gleason 1 and Gleason 2 data and export these as Gleason 3 (right side) records. Details about the NOTEXISTS, ADD and IF nodes’ semantics and why NOTEXISTS requires a second operand are given in Tables F and G in S1 File.

The *mapping ontology* connects the target ontology to the source ontology with manually created semantic relationships between medical concepts. We distinguish between simple and complex mappings. Simple mappings with a *hasImport* property express that the connected concepts share the same meaning (see Fig. 2, mapping (1)). Complex mappings are used whenever data transformation is required. In the mapping ontology, they are represented by intermediate nodes that express a filter operation or data transformation between the target node and exactly two operand nodes (e.g. *ADD_1*, see Fig. 2, mapping (2)). The different properties *hasOperand1* and *hasOperand2* allow the definition of non-commutative operations. Mapping nodes can be cascaded to full expression trees to support composed operations as shown in Fig. 4.

**Figure 5:**
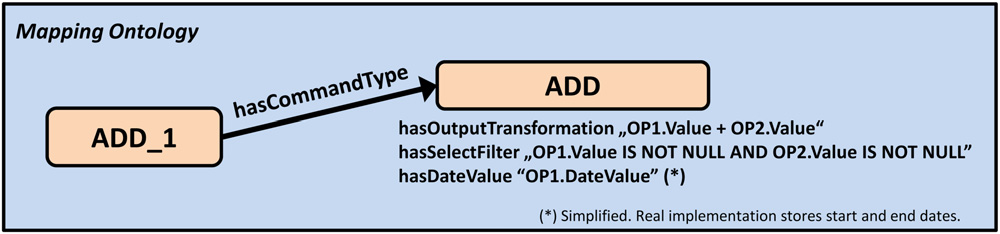
Command type definitions describe how to process mapping nodes from the mapping ontology. All intermediate nodes in the mapping ontology are connected to a command type definition. They contain SQL code fragments, which describe how to filter and transform the facts data derived from operands 1 and 2 (OP1 and OP2).

In addition, we have to define the processing method of complex mapping nodes. Fig. 5 shows once again the complex mapping node *ADD_1* from Fig. 2, which was used to define *Gleason Score 3* as the summation of the two operands *Gleason Score 1* and *Gleason Score 2.* It illustrates that *ADD_1* is connected to a command definition, *ADD*. The value of the *hasOutputTransformation* datatype property is an SQL database operation that adds the entries of the *Value* column of the two operand record sets OP1 and OP2 (as stated above, the *Value* column is part of the internal data model). The content of *hasSelectFilter* ensures that values for both operands exist. The *hasDateValue* property expresses that the time stamp for the result set (Gleason Score 3) has to be taken from OP1 (Gleason Score 1). The relevant ontology constructs, including the currently implemented operations, are shown in Tables E-J in S1 File.

### Second step of the automated process: Translation of the ontologies into SQL

**Figure 6:**
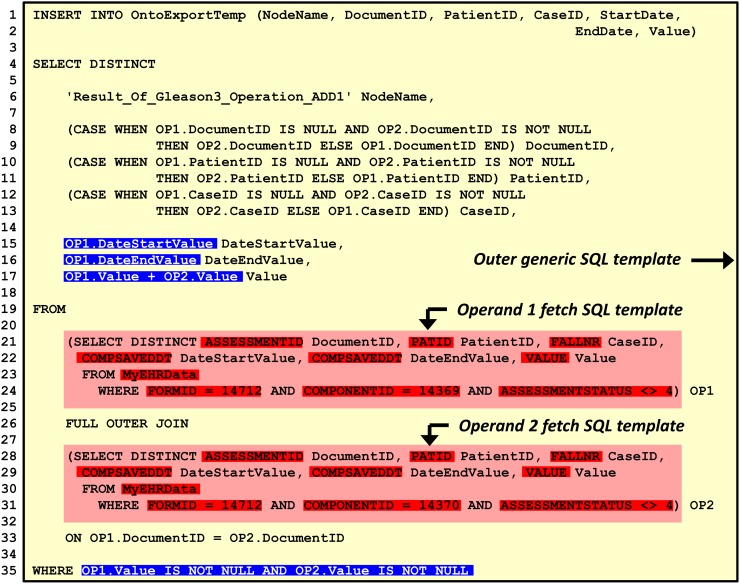
Software-generated SQL transaction that processes one intermediate mapping node. This real-world example shows the SQL code constructed from the example in Fig. 2 (complex mapping (2)). The inserted SQL fragments taken from the ontologies are printed in bold and highlighted in blue (how to transform the data) and red (how to access the data). The software that created this SQL code also generates the NodeName column entry in line 6.

A software component populates an SQL template with information stored in the ontologies. It generates an SQL statement for each mapping node in the mapping ontology. Fig. 6 shows a populated SQL template, which processes the mapping node *ADD_1* from Fig. 2 and stores the result in a temporary database table. The SQL template is the same for all node types, including arithmetic, relational and string processing operations (see Tables F-H in S1 File for additional examples). The statement initially fetches the data records for both operand nodes (result sets OP1 and OP2) according to the definition in the source ontology (lines 21–24 and 28–31). Both result sets are retrieved in an internal data model, which comprises six columns *DocumentID*, *PatientID*, *CaseID*, *DateStartValue*, *DateEndValue* and *Value*. The SQL statement joins both result sets on the entity (*DocumentID*, lines 26 and 33). This allows computations between data elements from the same form. To perform the data transformation of the mapping node, the statement applies the specified database operation (line 15–17) and filter (line 35), which were described in the *hasOutputTransformation* and *hasSelectFilter* properties. The result is written to a temporary database table (lines 1–2) that is also defined by the *hasSourceTable* property inside the ontologies.

Cascaded mapping networks (as shown in Fig. 4) are processed sequentially during the export. To find the next node, we apply a simple rule: A mapping node can only be processed if the data it accesses (both operands’ data records) are already available. All nodes inside the source ontology are considered to be ready for processing, because their data is already available in the source system’s database. During the export, the export software uses SPARQL queries [47] to find a random, but valid next node. SPARQL is a query language similar to SQL, but used for RDF ontologies. The export software creates an SQL script with one SQL statement as shown in Fig. 6 for each mapping node. When executing the script on the database, it automatically extracts, transforms and transfers the data records into the target database.

### Overloading internal data model properties with values from other data elements

A special mechanism allows replacing the values in the *DocumentID*, *PatientID*, *CaseID*, *DateStartValue*, *DateEndValue* columns of the internal data model with values from other concepts. This can be done by creating ontology statements that follow the convention *ConceptA hasXColumn ConceptB*, where *X* is one of *DocumentID*, *PatientID*, *CaseID*, *DateStartValue* or *DateEndValue*.

**Figure 7:**
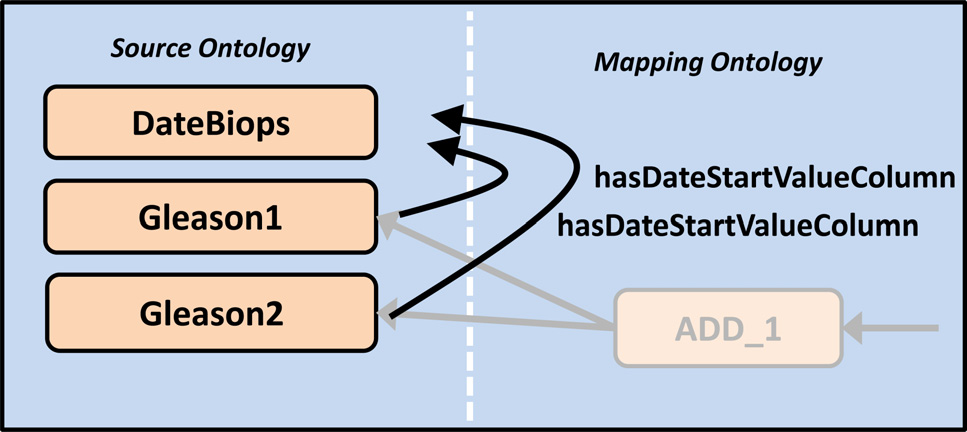
Overloading internal data model properties. This real-world example illustrates how semantic relationships between source data elements are stored explicitly in the ontologies and how they can be processed: Stating that *Gleason1 hasDateStartValueColumn DateBiops* e.g. tells the export software to use the data entry in the Value column of DateBiops as DateStartValue in Gleason1. Gleason2 is processed the same way.

This approach can deal with the time stamping problem that was shown in Fig. 1, where the correct time stamp of the data elements *Gleason1* and *Gleason2* was stored in a separate *DateBiops* field. Normally, the template-generated SQL code would use the storage date of the form for all data records. This is often acceptable under the assumption that the clinical documentation follows promptly the medical interventions and observations. However, in our example, a more timeliness data element *DateBiops* is available,which indicates the time when the biopsy was taken. By using the above-mentioned mechanism and by stating that *Gleason1 hasDateStartValueColumn DateBiops* and *Gleason2 hasDateStartValueColumn DateBiops* (see Fig. 7) the export software can replace the original operand-fetch SQL with other sub-selects. This in turn replaces the default *hasDateStartValueColumn* value (“2011–05–06”) with the *hasValueColumn* value of *DateBiops* (“2011–03–04”) of the data during the export.

Once such relationships have been defined in the source ontology, they are automatically considered in other mapping projects.

### Handling of missing and erroneous values

Our approach is also capable of dealing with erroneous and missing (“NULL”) values. In the example given above, the ADD node requires both operands to have existing data (see line 35 in Fig. 6 and the *hasSelectFilter* property in Fig. 5) because we specified that a Gleason Score 3 could not be calculated if one of the two operands is missing. However, we also defined “tolerant” node types, which explicitly allow one of the operands to have missing values. Depending on the operation, such NULL entries are replaced by the neutral element (0 or 1 for arithmetic operations, empty string for string operations). The use of tolerant or more stringent node types depends on the medical background of the mapping.

### Overview

**Figure 8:**
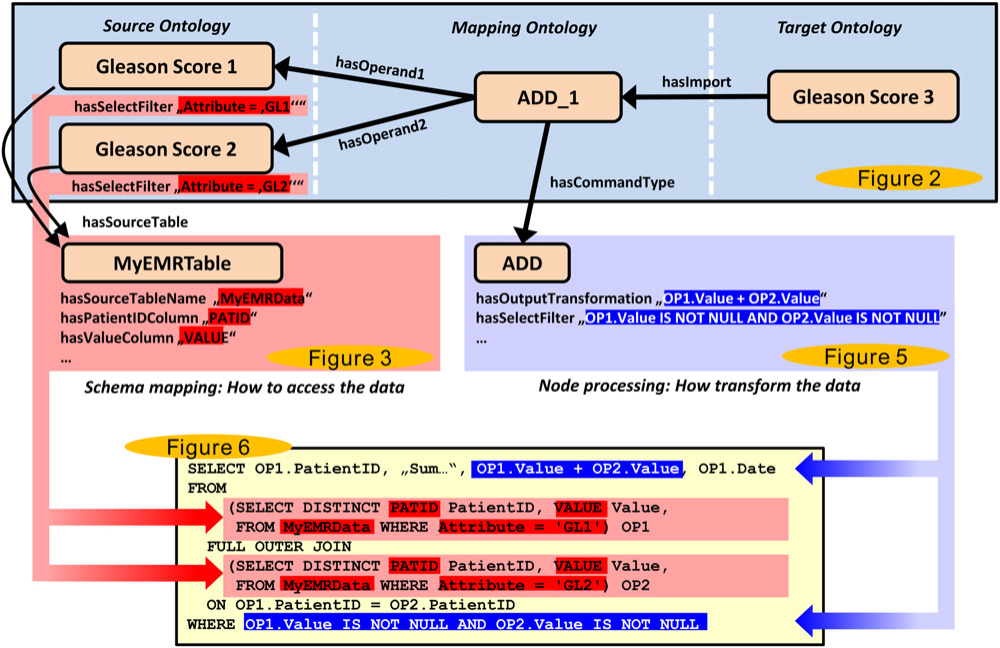
Overview of the approach. The illustration shows an overview of our approach by combining several of the previous figures in a simplified fashion. The upper part (blue box) represents a mapping, which is visible to the user. The parts in the middle are internal ontology concepts that are hidden for the user. The SQL code in the lower part has been automatically compiled from the above ontologies.

Fig. 8 provides an overview of the general approach by combining the information from the previous sections and figures. It shows how the information that is encapsulated in the ontologies (upper and middle part) is used to construct the SQL statement (lower part). The highlighted red parts represent the database schema mapping for the two operand nodes (Gleason Score 1 and 2) and describe how to perform the extraction of the source data, whereas the blue parts describe how to process the data (corresponding to the mapping).

### Generation of source ontologies

An important prerequisite for our system is the generation of the source ontology. There are different options, depending on the database schema and the complexity that is used to store the metadata (i.e. names of forms and data elements). If this metadata is available in an EAV-like format, an SQL script can be used to query the contents and to create the ontology triples (an example is available online).

For relational database schemas it is more difficult to access the metadata, because it is part of the database schema (i.e. column names). One could either model the ontology manually or make use of tools for metadata discovery (e.g. [48]).

An important feature of the source ontology is the syntactic separation between the source ontology concepts and the actual source data. Syntactic separation introduces an abstraction layer from the database schema, i.e. that the user no longer has to deal with the database structures and how the data is stored. Whenever the source system changes, the source ontology has to be modified to reflect these changes. For database schema changes (e.g. table or column names), a small modification of the routine that generates the source ontology can be sufficient. If the contents of the source system change, e.g. due to the versioning of data elements or the creation of new assessment forms, only the new data items have to be added.

### Implementation

**Figure 9:**
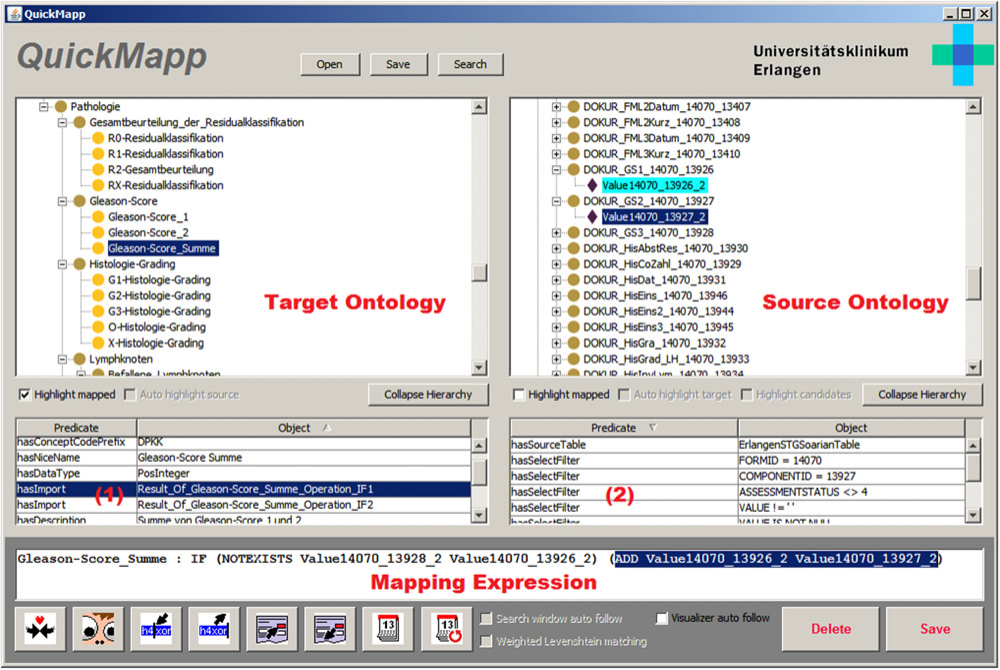
Screenshot of the QuickMapp tool. As indicated with the red notes, the tool shows the target ontology on the left and the source ontology on the right side. The statement browsers below the ontologies show the statements connected of the selected above concept, e.g. incoming mappings (1) or hasSelectFilter statements (2). Mappings can be created with a mapping expression editor in a bracketed prefix notation. The mapping shown is the same as in Fig. 4. Without the EMR-specific local naming, the statement would be: Gleason3: IF (NOTEXISTS Gleason3 Gleason1) (ADD Gleason1 Gleason2).

The whole approach has been implemented as a set of Java tools called “OntoImportSuite” using NetBeans (http://netbeans.org) and the Apache Jena framework (https://jena.apache.org/). It comprises an ontology editor (OntoEdit) for editing of i2b2-specific target ontologies, a manual mapping application (QuickMapp, see Fig. 9) and an OWL-to-SQL processor (OntoExport).

## Results

### Creation of a Soarian EMR source ontology

We created a comprehensive source ontology for the complete Soarian EMR of the Erlangen University hospital, which comprises 28,840 classes (with 785 first-level classes denoting the forms and 28,055 second-level classes denoting the EHR data elements) and 69,841 instances representing the values, including versioning. In addition, the ontology contains what we call “abstract concepts”. These are ontology concepts that do not describe original data in the source system but rather information that was derived from these original data. For example, we created concepts that permit to discover patients for whom a given documentation form has been created, independently whether the form was completed or not. Thus we may e.g. detect cancer patients in our database, because a radiotherapy form exists.

### Data integration in a cross-institutional setting

In a multicenter research scenario we faced the task to build a shared tissue sample database [34]. The target data set comprised elements such as: *clinical study participation, clinical and pathological TNM, time between diagnosis and surgery, Gleason scores* and many more. This dataset has been modeled as an OWL target ontology with 51 classes and 189 leafs using the OntoEdit tool.

We then mapped this target ontology to our EMR source ontology. In this case we identified 143 relevant data elements from 20 different forms such as anamnesis, tumor board report and therapy reports.

The mapping ontology for this scenario comprises 648 mappings including 50 complex mappings (see Table 1 for the use of complex operators). To identify patients with a Gleason Score greater two, we added eight custom string values to deal with such classifications. In the mentioned example the custom string value “2” has been used to export data only for patients with a Gleason Score greater two. Other operators listed in Table 1, such as GREATERVT, EXISTS or NOTEXISTS were required for either temporal selections of the appropriate first or last data value respectively for selecting patients with the correct type of cancer.

**Table 1:** Types and numbers of intermediate nodes that were used in complex mappings in the urology project.

**Node type**	**Times used**	**Description**
ADD	9	Adds the numeric values of both operands
EQUALS	2	Returns ‘TRUE’ if operand 1 = operand 2
GREATER	2	Returns ‘TRUE’ if operand 1 > operand 2
GREATERVT	32	Returns ‘TRUE’ if operand 1 > operand 2, operand 1 can be NULL
EXISTS	26	Returns ‘TRUE’ if operands 1 and 2 exist (with operand 1 = operand 2)
NOTEXISTS	8	Returns ‘TRUE’ if operand 1 does not exist and operand 2 exists (see Table G in S1 File for detailed explanation why operand 2 is necessary)
IF	64	Returns the value from operand 2 if operand 1 has the value ‘TRUE’

The named scenario is in continuous use since 2010. Thus, the SQL statements have been used to fill the research database of currently 529 patients and 21,878 observation facts. Changes in the EMR have been successfully addressed by updating the source and mapping ontologies.

In this scenario we also integrated source data of the clinical information system from another University hospital. In that case the EMR comprised effectively 3 fields for the Gleason score, namely Gleason 1, Gleason 2 and the summary Gleason. To perform this task we implemented an additional source and mapping ontologies but reused the same target ontology.

## Discussion

### Overcoming the challenges of accessing and processing heterogeneous EMR data

This paper presents a novel approach for the ontology-based integration of heterogeneous medical data between clinical and research databases. It makes heavy use of abstraction by shifting the database-centered, technical thinking based on tables, columns and rows to a focus on medical concepts and their relations. The structural view of the data source, the data target and the mapping undergo an explicit externalization within three ontology constructs.

Our approach does not eliminate the mapping effort itself, although we strive to make the mapping more sustainable. Relying on machine-processible Semantic Web standards, our proposed method enables the re-use of the captured mapping knowledge to support, for example, the automated SQL code generation for the physical ETL process from one system to another.

Our methodology could act as an extension for research databases by providing a universally machine-readable, semantic ETL framework with a fine granularity that reaches down to the level of versioned value sets in the source systems. We have shown that our approach is compatible with i2b2 and that it is capable of processing highly heterogeneous EMR assessment form data. Furthermore, this method provides solutions for the challenges that were originally mentioned in the introduction:

**A) Our method supports the mapping of non-standardized data elements to standard terminologies.** Custom data elements are typical features of modern EMR systems that support the definition of customized clinical documentation forms. For secondary use research projects it is vital to standardize these data, e.g. by mapping them to standard terminologies. In our approach, these mappings form part of the permanent and reusable mapping ontology. We currently map to custom domain ontologies (e.g. the one described in section 3.2), but mappings to standard terminologies are possible. Some nomenclatures, such as SNOMED-CT, post-coordinate medical concepts. This means that one concept is actually a composition of others [49]. We support the integration of such concepts by making use of complex one-to-many or many-to-one mappings, because they allow the arbitrary merging, splitting and logical linking of concepts. Thus mappings to ontologies such as the NCI Thesaurus [50] or SNOMED-CT are possible. Once more terminologies become available in OWL (see e.g. [51, 52]) it will be easier to store and maintain such mappings in this format.

**B) Our system provides knowledge management functions for EMRs.** By manually mapping semantically equal concepts from the source system to single concepts in the target ontology, the users of our system create a verified, machine-processible knowledge repository, which is similar to a medical data dictionary. Due to the manual mapping process and the use of intermediate nodes, it is possible to explicitly define the semantic relationships between similar data elements, whereas others (especially automated mapping methods) are limited to only describing the level of similarity (e.g. [53]).

Apart from the automated SQL code generation for the ETL process, the ontologies of our approach can be post-processed and queried for other purposes as well, e.g. to derive provenance information of data. The mapping ontology can be evaluated in terms of node types, performed transformations and filter mechanisms used, as shown in Table 1. In conventional ETL tools, such identification would be very difficult if not impossible.

The provenance information is useful for the maintenance of the source system. It can be evaluated in order to identify redundant data elements, which is typically the case if two or more source system concepts are mapped to a single concept in the target ontology. When creating new content in a source system, e.g. when a new EMR form has to be created, a quick look-up in the source ontology enables the identification of already existing data elements. This avoids the accumulation of inconsistent concept naming and value sets because already existing data elements can be reused.

**C) Our approach provides means for the semantic annotation of EMR systems.** We recreate and preserve contextual medical relationships between data elements within the mapping ontologies. This comprises also medical knowledge that may be hidden within the EMR. An example has been given in Fig. 1. The mentioned pathology form contains the hidden medical knowledge that Gleason is a compound score with two components, which refer to the same date of biopsy. Our mapping ontology makes this explicit.

**D) Our approach facilitates data integration between institutions.** The target ontology may be shared between several institutions. Every institution can define its own source ontology and mappings to the target ontology. The generated SQL statements then perform the data extraction and processing. Although we cannot eliminate semantic gaps between source and target, we are able to model individual as well as reusable scenarios to deal with such gaps in a formalized and reproducible fashion.

### Related research

Current state-of-the-art single source research platforms such as *Informatics For Integrating Biology And The Bedside* (i2b2) [46], the *Shared Health Research Network* (SHRINE) [26] or *Electronic Health Records For Clinical Research* (EHR4CR) [30] use a data warehouse approach. Such data warehouses are based on common information models and allow the storage of heterogeneous medical data. To transfer clinical data into a research data warehouse an ETL process is required to extract and transform data from a clinical source system and to load it. The usual approach comprises copying table structures from the clinical system to a staging area, transforming them to a given target structure with the help of a mapping or ETL tool and to finally load the source system contents into the data warehouse. The complete mapping process remains more or less hidden within the respective ETL tool. In contrast, we make both the structure of source and target system and the mapping explicit in reusable triple structures within the ontologies.

Some data warehouses permit, similar to our approach, the automated generation of SQL statements (e.g. [54–63]). Upon a first glance, our implementation shares several similarities with these tools. They all feature the abstract and often graphical modeling of ETL jobs, which are then automatically translated into SQL code or another representation that processes the data. The popular Talend Open Studio ETL software [56] for example generates Java code. Furthermore, they contain useful features such as error tracking and volume auditing. However, these modeled ETL jobs are specific to the respective ETL software and do not permit external processing. In comparison, our approach uses machine-processible ETL definitions that can be reused outside the ETL environment. The advantage is that thus we can e.g. support sustainable mapping to external terminologies (see chapter 4.1) as well as external statistics of the mapping effort and mapping performance.

Bache et al. [30] describe how they connect to different DWHs using an SQL-template-based query mechanism in order to achieve a mapping from their source system to the data model of the EHR4CR platform. The use of predefined SQL queries is similar to our approach. However, while Bache et al. use different static queries for different medical data categories, our approach permits the use of dynamic templates attached to each medical concept. We extend this feature down to the value level using unique *hasSelectFilter* properties for the templates.

The development of ETL jobs for heterogeneous data is a difficult task and some researchers aim to partially automate it. The research area of *schema matching* and *mapping* develops algorithms that try to find correspondences between two different database schemas [64, 65]. For example, Sun’s MEDIATE [53], which also uses semantic networks to store mappings between semantically equivalent concepts in different databases, is such an automatic schema matcher. The system is also capable of automatically creating SQL code for data retrieval by including “database links” into the semantic network. It is worth noting that such matching methods cannot create complex mappings which support data transformations between multiple concepts. To our knowledge, no such implementation exists yet, and we believe it would be very challenging to develop one in the case of uncoded EHR data, due to its extreme heterogeneity.

Others propose the use of Semantic Web technologies [37] to ease the challenge of heterogeneous data integration. In most implementations the complete, originally relational research data is made available in RDF triples [27, 66–70]. In this context, tools such as D2RQ [71] or Quest [72] have been developed and have been used e.g. in [73]. Such on-the-fly conversions, however, do not ease the challenges of reusing the intrinsic EMR data and the semantic annotation of the EMR, because the generated RDF is almost an exact copy of the original database schema ([37], p.345). This means that that original data is only represented in a different syntax (triples), but with no semantic value added. To continue to work with such data, technologies such as SPARQL [47] would have to be used in the same way as SQL for relational databases, and a semantic integration would have to take place afterwards. We believe it is better to convert the metadata of the source system to RDF, separated from the facts data. This allows a flexible representation and modeling of local specialties, such as the data element versioning and can be used to provide a true semantic mapping between source and target systems.

### Integration with conventional ETL environments

The generated SQL code automatically handles the extraction, transformation and loading of the mapped data elements into i2b2. ETL methodologies for data warehouses can be considerably complex (e.g. [74, 75]), and depending on the character of the data different tools are used. Our proposed method could complement conventional data warehousing setups by simplify the integration of highly heterogeneous medical data, such as EMR assessment form data. In such a case the generated SQL would become a parallel track in the transformation pipeline. With the generated SQL scripts integrated into commercial or free ETL solutions (e.g. [54–63]), the approach would also benefit from error tracking, volume auditing and other features.

### Portability to other institutions and environments

The proposed semantic ETL method can be transferred to another environment or research institution, provided that this institution has access to the metadata of its EMR database:

1. As described in 2.4, a process is required that generates the source ontology for the source system. For EMRs with relational EAV-like databases, this can be achieved with SQL scripts.
2. The manual mapping process, which may be supported with the QuickMapp tool, must be performed to define mappings and conversions between source and target ontology items.
3. The OntoExport tool reads all information from the source, target and mapping ontology and automatically produces the SQL to extract and transform the required data items to the target system.

While our approach is generic and should work with any relational database system, our OntoExport tool currently generates SQL code for Oracle. By modifying the SQL code fragments in one of the ontologies it is possible to generate SQL code compatible with other SQL-based database systems (see Fig. 5, *OntoMappingSystem.owl*). Besides Oracle we have also tested Microsoft SQL-Server.

### Limitations

The creation of some complex mappings is inconvenient in our approach. We discussed mappings between different surgical interventions and body parts for which these interventions could be applied. This would have resulted in 108 rather ineffective partial mappings, because we do not yet support mappings at hierarchical levels, e.g. between the class of all interventions and the class of all body parts.

Today, our supported target system is i2b2. Thus, the current implementation incorporates some i2b2-specific features related to the semantics of the target ontology and the internal data model of the facts data. While the generic and pragmatic i2b2 system offers extensive research capabilities, additional standardization would simplify the data export to other research platforms. It might even enable us to develop our approach towards a comprehensive semantic data integration software suite. Development towards the ISO/IEC 11179 MDR metadata repository standard [76], openEHR archetypes [77], HL7 RIM [78], ISO 13606 [79] or the CDISC standards [80] could be a future task. Even less complex standards such as SKOS [81] could be beneficial, as shown in [82, 83]. Complying with such standards would simplify interfacing with non-i2b2 systems and the adaption to other sites.

A current practical limitation of our system is the storage of ontological knowledge in local OWL files. Therefore target ontologies must be copied between institutions even if they are identical. Switching to a central triple store or a terminology server, such as LexEVS [84], would remedy this issue.

## Outlook and Future Research

We have presented a novel approach for semantic ETL in single source projects. Future work should concentrate on standardizing the target ontology and internal data model as well as the integration of additional mappings towards standardized terminologies, such as the NCI Thesaurus or SNOMED CT. Additional research concerning the ontological modeling of advanced properties within assessment forms will be necessary, e.g. to enable the creation of hierarchical and other abstract relationships between different form elements.

### Availability of the software (OntoImportSuite)

The software and source code (licensed under the GPL3) are available on GitHub (https://github.com/sebmate/OntoImportSuite). In addition we have supplied a demonstration subset of the diverse ontology contents, which is needed to replicate the methodology and the SQL script that was used to generate the source ontology for our Soarian EMR system as described in this paper. The installation requires an i2b2 1.6.x instance or database schema. Please note that the software is of prototypical character and provided “as is”, without any warranties.

## Supporting Information

S1 File Appendix containing Tables A-J. **Table A.** Classes of the ontology MDR-System.owl. **Table B.** Instances of class MDR-DataType in MDR-System.owl. **Table C.** Datatype properties of the ontology in MDR-System.owl. **Table D.** Object properties of the ontology in MDR-System.owl. **Table E.** Class hierarchy of the ontology OntoMappingSystem.owl. **Table F.** Instances of the class ArithmethicOperation in the ontology OntoMappingSystem.owl. **Table G.** Instances of the class RelationalOperator in the ontology OntoMappingSystem.owl. **Table H.** Instances of the class StringOperation in the ontology OntoMappingSystem.owl. **Table I.** Object properties of the ontology OntoMappingSystem.owl. **Table J.** Datatype properties of the ontology OntoMappingSystem.owl.
(DOCX)
