# Correction: Ontology-Based Data Integration between Clinical and Research Systems

**DOI:** 10.1371/journal.pone.0122172

**Published:** 2015-03-23

**Authors:** The PLOS ONE Staff

The data availability statement for this paper is incorrect. Please view the correct statement here.

The software and source code (licensed under the GPL3) are available on GitHub https://github.com/sebmate/OntoImportSuite). In addition we have supplied a demonstration subset of the diverse ontology contents, which is needed to replicate the methodology and the SQL script that was used to generate the source ontology for our Soarian EMR system as described in this paper.

## References

[1] Mate S, Köpcke F, Toddenroth D, Martin M, Prokosch H-U, Bürkle T, et al (2015) Ontology-Based Data Integration between Clinical and Research Systems. PLoS ONE 10(1): e0116656 doi: 10.1371/journal.pone.0116656 25588043 10.1371/journal.pone.0116656 PMC4294641

