# Supplementary material for: Ontology-Based Data Integration between Clinical and Research Systems
Source: PLoS One. 2015 Jan 14;10(1):e0116656. doi: 10.1371/journal.pone.0116656 (PMC4294641; doi:10.1371/journal.pone.0116656)
Supplement: S1 File — Table A. Classes of the ontology MDR-System.owl. Table B. Instances of class MDR-DataType in MDR-System.owl. Table C. Datatype properties of the ontology in MDR-System.owl. Table D. Object properties of the ontology in MDR-System.owl. Table E. Class hierarchy of the ontology OntoMappingSystem.owl. Table F. Instances of the class ArithmethicOperation in the ontology OntoMappingSystem.owl. Table G. Instances of the class RelationalOperator in the ontology OntoMappingSystem.owl. Table H. Instances of the class StringOperation in the ontology OntoMappingSystem.owl. Table I. Object properties of the ontology OntoMappingSystem.owl. Table J. Datatype properties of the ontology OntoMappingSystem.owl. (DOCX) [file pone.0116656.s001.docx]

**Appendix For Article

*Ontology-Based Data Integration
Between Clinical and Research Systems***

This appendix fully describes the ontology constructs that are used in the source, mapping and target ontologies. All three ontologies import other ontologies (*MDR-System.owl* and *OntoMappingSystem.owl*) by making use of the *owl:imports* construct. The target and source ontologies typically contain hierarchies (created with *rdfs:subClassOf* statements) to arrange the concepts; the classes or instances are then linked to the concepts below. *Note: Dashes are used to indicate the class hierarchy level.*

## MDR-System.owl

This ontology contains classes and properties that are to be used for the description of target ontologies. The contained information is used to generate the i2b2 ontology.

Used name space: mdr: http://www.uk-erlangen.de/MDR-System#

| **Class** | **Description** |
| --- | --- |
| MDR-Context | Class of all context instances, which can be assigned to the sub-classes of *MDR-Dataelement* by using the property *hasContext*. |
| MDR-Dataelement | Root class of the full concept hierarchy. All sub-classes of this are used to generate the i2b2 ontology. |
| MDR-Unit | Class to describe units and associated conversion factors. |
| MDR-DataType | Class of all data types supported by i2b2 (see Table B). |

**Table A:** Classes of the Ontology MDR-System.owl.

| **Instance** | **Description** |
| --- | --- |
| Enum | Enumeration |
| Float | Floating point value |
| Integer | Integer value |
| PosFloat | Positive floating point value |
| PosInteger | Positive integer value |
| String | String value |

**Table B:** Instances of class MDR-DataType in MDR-System.owl.

| **Property** | **Description / Mapping** |
| --- | --- |
| i2b2OntoDatatypeProperty | Parent property of all datatype properties that are used to describe the i2b2 ontology. |
| - hasDescription | Content for the i2b2 ontology column "c_tooltip" |
| - hasFlagsToUse | Content for the i2b2 ontology XML attribute <Flagstouse> |
| - hasNiceName | Content for the i2b2 ontology column "c_name" |
| - i2b2ConceptCodeProperty | Parent property of all datatype properties that are used to describe i2b2 concept codes. |
| - - hasConceptCodePrefix | Prefix of the i2b2 concept code in the column "c_basecode" (prefix:suffix) |
| - - hasConceptCodeSuffix | Suffix of the i2b2 concept code in the column "c_basecode" (prefix:suffix) |
| - i2b2LabValueProperty | Parent property of all datatype properties that describe lab values |
| - - hasLowOfLowValue | Contents of the XML attribute <LowofLowValue> |
| - - hasHighOfLowValue | Contents of the XML attribute <HighofLowValue> |
| - - hasLowOfHighValue | Contents of the XML attribute <LowofHighValue> |
| - - hasHighOfHighValue | Contents of the XML attribute <HighofHighValue> |
| - i2b2MedicationProperty | Parent property of all datatype properties that describe medications |
| - - hasLowOfToxicValue | Contents of the XML attribute <LowofToxicValue> |
| - - hasHighOfToxicValue | Contents of the XML attribute <HighofToxicValue> |

**Table C:** Datatype properties of the ontology in MDR-System.owl.

| **Property** | **Description** |
| --- | --- |
| hasContext | Can be used to assign an instance of the *MDR-Context* class to an instance of the *MDR-Dataelement* class. |
| hasDataType | Assigns an instance of the *MDR-DataType* class to an instance of the *MDR-DataType* class. It is also used to create the contents of the XML attribute <DataType> in the i2b2 ontology. |
| hasUnits | Assigns an instance of the *MDR-Unit* class to an instance of the *MDR-DataType* class. It is also used to create the contents of the XML attribute <NormalUnits> in the i2b2 ontology. |

**Table D:** Object properties of the ontology in MDR-System.owl.

## OntoMappingSystem.owl

This ontology contains classes and properties that are to be used for the description of mapping and source ontologies.

Used name space: omsys: http://www.uk-erlangen.de/OntoMappingSystem#

| **Class** | **Description** |
| --- | --- |
| DatabaseConnection | Class of all database connections |
| SourceTable | Class of all database tables in the source system |
| OperationCommand | Class all intermediate node types |
| - ArithmeticOperation | Arithmetic operations |
| - RelationalOperator | Relational operators |
| - StringOperation | String operations |
| StatusTypedItem | Class of all source ontology items and intermediate nodes |
| - UnprocessedItem | Class that contains all unprocessed intermediate nodes. After processing, they are moved to class *ProcessedItem*. |
| - ProcessedItem | Class that contains all processed intermediate nodes |
| - StringItem | Class for intermediate nodes that store arbitrary strings |

**Table E:** Class hierarchy of the ontology OntoMappingSystem.owl.

The following tables show the intermediate node types that have been implemented so far. They are stored as instances of the class *OperationCommand*. The columns "T" and "VT" describe whether a "tolerant" and/or "very tolerant" operation is also available (e.g. "ADDT" and "ADDVT" for "ADD"). The tolerant variants allow NULL values in operand 1; the very tolerant ones allow having a NULL value in either operand 1 or 2. Unless otherwise specified, all nodes return the entity attributes (DocumentID, PatientID, CaseID, DateStartValue, and DateEndValue) of operand 1 by default.

| **Operator** | **T** | **VT** | **Description** |
| --- | --- | --- | --- |
| ADD | X | X | Adds both operands |
| SUBTR | X | X | Subtracts operand 2 from operand 1 |
| MULT | X | X | Multiplies operand 1 with operand 2 |
| DIV | X |  | Divides operand 1 through operand 2 |
| DIFF |  |  | Calculates the difference between both operands |

**Table F:** Instances of the class ArithmethicOperation in the ontology OntoMappingSystem.owl.

| **Operator** | **T** | **VT** | **Description** |
| --- | --- | --- | --- |
| EQUALS | X | X | Returns 'TRUE' if operand 1 = operand 2 |
| GREATER | X | X | Returns 'TRUE' if operand 1 > operand 2 |
| GREATEREQUAL | X | X | Returns 'TRUE' if operand 1 >= operand 2 |
| LESSER | X | X | Returns 'TRUE' if operand 1 < operand 2 |
| LESSEREQUAL | X | X | Returns 'TRUE' if operand 1 <= operand 2 |
| EXISTS |  |  | Returns 'TRUE' if operands 1 and 2 exist (Typically operand 1 = operand 2) |
| NOTEXISTS |  |  | Returns 'TRUE' if operand 1 does not exist and operand 2 exists (The existence of a reference node in operand 2 is necessary, because it is impossible to check for absence of values in documents, e.g. in EMR forms. If the system's database schema is EAV-like, absent values are simply not being stored, therefore this reference is necessary.) |
| IF |  |  | Returns the value from operand 2 if operand 1 has the value 'TRUE' |

**Table G:** Instances of the class RelationalOperator in the ontology OntoMappingSystem.owl.

| **Operator** | **Description** |
| --- | --- |
| INSTR | Returns 'TRUE' if operand 1 is a substring of operand 2 |
| STRPOS | Returns the position of operand 2 inside operand 1, operand 1 is a substring of operand 2. If not, nothing is returned. |
| STRPOS2 | Returns the position of operand 2 inside operand 1, operand 1 is a substring of operand 2. If not, '0' is returned. |
| LCASE | Returns the value from operand 1 in lower case |
| UCASE | Returns the value from operand 1 in upper case |
| HEAD | Returns the partial string from operand 1, which starts at the first character and ends at the position stored in operand 2 |
| TAIL | Returns the partial string from operand 1, which starts at the position stored in operand 2 and ends at the last character |

**Table H:** Instances of the class StringOperation in the ontology OntoMappingSystem.owl.

| **Property** | **Description** |
| --- | --- |
| hasDatabaseConnection | Assigns a database connection instance of the class *DatabaseConnection* to the fact table of the class *SourceTable* |
| hasSourceTable | Assigns leaf element from the source ontology or a mapping node from the mapping ontology a fact table instance of the class *SourceTable* |
| hasCommandType | Assigns a command type instance from the class *OperationCommand* to a mapping node in the mapping ontology. |
| hasImport | Assigns a mapping node from the mapping ontology or a lead element from the source ontology to a leaf element of the target ontology. |
| hasOperand | Property to express that a mapping node from the mapping ontology has a different node as operand. |
| - hasOperand1 | Makes a mapping node from the mapping ontology the first operand |
| - hasOperand2 | Makes a mapping node from the mapping ontology the second operand |

**Table I:** Object properties of the ontology OntoMappingSystem.owl.

| **Property** | **Description** |
| --- | --- |
| CommandType | Properties that contain SQL snippets that used in the SQL statements which construct new result records |
| - hasDateStartValue | Describes from which operand the start timestamp of a diagnose should be taken from |
| - hasDateEndValue | Describes from which operand the end timestamp of a diagnose should be taken from |
| - hasOutputTransformation | Contains the database operation to process operand 1 with operand 2 |
| - hasSelectFilter | Contains a filter statement that is used in the WHERE clause |
| DatabaseConnectionProperty | Defines a database property (currently, only Oracle is supported) |
| - hasHostName | Defines the host name of the database server |
| - hasUserName | Defines the user's login |
| - hasPassword | Defines the user's password |
| - hasPort | Defines the network port |
| - hasSID | Defines the Oracle SID |
| TableDescriptionProperty | Describes information that is used to access a database fact table |
| - hasSourceTableName | Defines the table's name |
| - hasAccessSQL | Contains a generic SQL block that can be used to process two operand nodes. The block contains variables that are replaced by OntoExport. |
| - hasOperandFetchSQL | Contains a generic SQL block that fetches the data records one operand |
| - hasDocumentIDColumn | Defines the column name that stores the document ID |
| - hasPatientIDColumn | Defines the column name that stores the patient ID |
| - hasCaseIDColumn | Defines the column name that stores the case ID |
| - hasDateStartValueColumn | Defines the column name that describes the start date time stamp of an observation fact |
| - hasDateEndValueColumn | Defines the column name that describes the end date time stamp of an observation fact |
| - hasValueColumn | Defines the column name that contains the actual value |
| hasStringValue | Assigns a string data value to a string mapping node of class *StringItem* |
| isResultOfExpression | Assigns a processed partial expression in QuickMapp syntax to an intermediate node for debugging purposes. |

**Table J:** Datatype properties of the ontology *OntoMappingSystem.owl*.
